# Engineering actinomycetes for biosynthesis of macrolactone polyketides

**DOI:** 10.1186/s12934-019-1184-z

**Published:** 2019-08-13

**Authors:** Dipesh Dhakal, Jae Kyung Sohng, Ramesh Prasad Pandey

**Affiliations:** 1 Department of Life Science and Biochemical Engineering, Sun Moon University, 70 Sunmoon-ro 221, Tangjeong-myeon, Asan-si, 31460 Chungnam Republic of Korea; 2 Department of Pharmaceutical Engineering and Biotechnology, Sun Moon University, 70 Sunmoon-ro 221, Tangjeong-myeon, Asan-si, 31460 Chungnam Republic of Korea

**Keywords:** Macrolides, Biosynthesis, Polyketide synthase, Biosynthetic gene clusters, Metabolic engineering, Actinomycetes

## Abstract

Actinobacteria are characterized as the most prominent producer of natural products (NPs) with pharmaceutical importance. The production of NPs from these actinobacteria is associated with particular biosynthetic gene clusters (BGCs) in these microorganisms. The majority of these BGCs include polyketide synthase (PKS) or non-ribosomal peptide synthase (NRPS) or a combination of both PKS and NRPS. Macrolides compounds contain a core macro-lactone ring (aglycone) decorated with diverse functional groups in their chemical structures. The aglycon is generated by megaenzyme polyketide synthases (PKSs) from diverse acyl-CoA as precursor substrates. Further, post-PKS enzymes are responsible for allocating the structural diversity and functional characteristics for their biological activities. Macrolides are biologically important for their uses in therapeutics as antibiotics, anti-tumor agents, immunosuppressants, anti-parasites and many more. Thus, precise genetic/metabolic engineering of actinobacteria along with the application of various chemical/biological approaches have made it plausible for production of macrolides in industrial scale or generation of their novel derivatives with more effective biological properties. In this review, we have discussed versatile approaches for generating a wide range of macrolide structures by engineering the PKS and post-PKS cascades at either enzyme or cellular level in actinobacteria species, either the native or heterologous producer strains.

## Background

Natural products (NPs) from plants or microorganism, native or structurally modified have been utilized for the treatment of infections and ailment of disease conditions [1, 2]. Among all microorganisms, actinobacteria isolated from terrestrial or marine sources are characterized prominent producer of such NPs of pharmaceutical values, such as antibacterial, anticancer agents, anti-parasite, and immunosuppressant [3–5]. Actinobacteria are Gram-positive filamentous bacteria containing a high G+C content. They generally produce mycelium and reproduce by sporulation similar to filamentous fungi. However, they possess prokaryotic nucleoid and peptidoglycan cell wall significantly different from fungi [6]. Hence, the name “actinobacteria” is derived from Greek whereas; “aktis or aktin” meaning “ray” and “mukes” meaning “fungi”. The production of NPs from these actinobacteria is associated with diverse biosynthetic gene clusters (BGCs) present in these microorganisms. Each BGC contain a defined set of genes sufficient for biosynthesis of particular chemical structure. The majority of these BGC include polyketide synthase (PKS) or non-ribosomal peptide synthase (NRPS) or combination of PKS and NRPS.

**Fig. 1:**
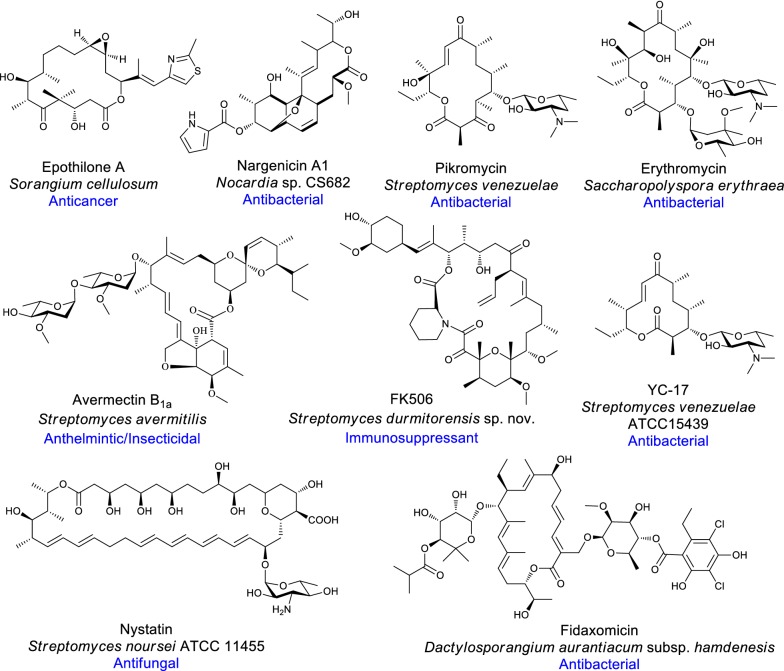
Structures of different macrocyclic compounds with their primary producer strain and potential use of these molecules

Macrolides include diversified chemical structures containing a core macro-lactone ring (aglycone) decorated with diverse functional groups, most commonly deoxy-sugars and amino-sugars [7]. They are biologically important for their uses in therapeutics as antibiotics (erythromycin, pikromycin, nargenicin, oleandomycin), anti-tumor agents (epothilone), immunosuppressant (rapamycin, FK506) and anti-parasites (avermectin) (Fig. 1) [8, 9]. Macrolides are categorized into different groups according to the number of atoms in the lactone ring, for example, 12-, 14-, 15-, 16-, 17-(ivorenolide B) or 18-(tedanolide C) membered macrolides [10]. They are generally biosynthesized by type I polyketide synthase (PKS) and modified by tailoring enzymes such as glycosyltransferase (GT), methyltransferase (MT) and oxidation enzymes as monooxygenase (MO), cytochrome P450 (CYP450) and oxidoreductase (OR).

The alarmingly increase in drug-resistant bugs or drug-tolerant cancer conditions have vitalized the need for bioactive molecules with new or improved pharmacological properties. Therefore, there is immersing interest on isolation and characterization of novel macrolides. The clinically useful macrolides have been traditionally derived from the screening of natural sources, and still the isolation of novel macrolides and evaluating their bioactivities has been an active area of research [11]. Most of the NPs, including macrolides, exhibit a broad range of pharmacophore, but still, they need to be structurally modified to ameliorate their chemical and biological properties for clinical uses [5, 12]. The total synthesis of macrolide derivative or chemical modification to generate semi-synthetic derivative have been an important tool for the development of novel molecules with enhanced pharmacological activities. However, such chemical approaches have limitations as many sites on the macrolactone ring and/or the sugar moiety are not amenable to desired chemical modifications [9, 13]. Another promising approach has been the biological engineering of the production hosts by rational metabolic engineering and synthetic biology tools. The major advantage of such biological process over chemical approach is cost-effectiveness, environmental friendliness, and easy scale-up possibilities [14, 15].

In this review, the progress on engineering of actinomycetes for deriving novel macrolides by engineering of aglycon biosynthesis and post-PKS tailoring enzymes has been summarized. The front part of the review includes early approaches of precursor-directed biosynthesis and mutasynthesis. Finally, the application of the advanced synthetic biological tools assisted by different “–omics” based approaches for combinatorial biosynthesis of macrolides have been discussed.

## Outline of biosynthesis of macrolides

**Fig. 2:**
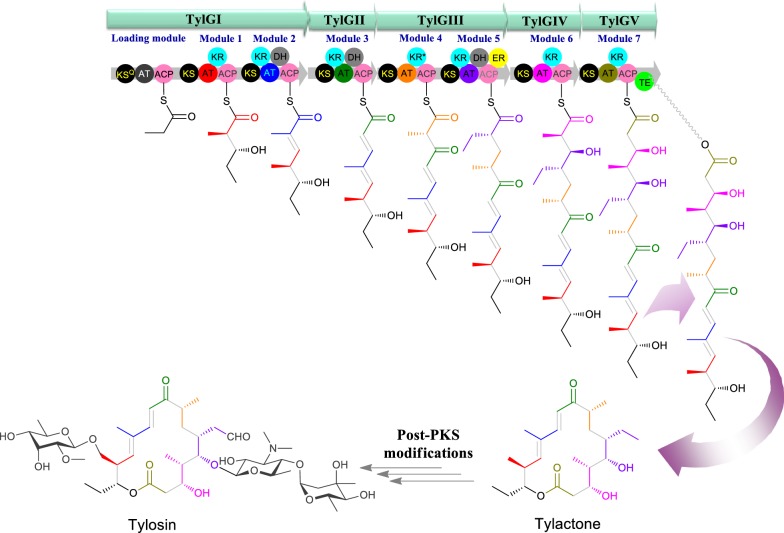
Biosynthetic gene assembly of tylosin biosynthesis gene cluster illustrating condensation and modification of different extender units to form tylactone aglycone which is further decorated with different post-modification enzymes to form tylosin

The type I PKS generally consist of multifunctional, multi-modular enzymes with non-iterative catalysis of one cycle of polyketide chain elongation. These enzymes are responsible for successive condensation of activated coenzyme A (CoA) thioesters (most commonly acetyl-, propionyl-, malonyl- or methylmalonyl-CoA) for such polyketide chain elongation. Each module contains a set of functional domains of acyl carrier protein (ACP), acyltransferase (AT), and, β-ketoacyl synthase (KS), which are essential for polyketide elongation [16, 17]. The CoAs used as substrate is selected and activated by an acyl transferase (AT) domain and transferred to the 4′-phosphopantetheinylated acyl carrier protein (ACP) domain. The ketosynthase (KS) domain catalyzes the decarboxylative Claisen-like condensation between the substrate and the growing polyketide, to form a carbon–carbon bond between the alpha carbon of the extender unit and the thioester carbonyl of the ACP-bound acyl chain [9, 18, 19]. Besides these minimal domains, additionally, there are β-keto processing domains, ketoreductase (KR), dehydratase (DH), and enoyl reductase (ER) that act sequentially to reduce the β-keto group into a fully saturated acyl chain [20]. The synthesized polyketide is off-loaded by thioesterase (TE) domain [21, 22]. Figure 2 shows the schematic organization of modular PKS as exemplified by tylosin (Tyl) PKS, which is 16-membered macrolide produced by *Streptomyces fradiae*. The tylosin PKS includes one loading module and seven extension modules terminating in thioesterase (TE) domain to generate tylactone [23–25]. The aglycone is produced by the cordial action of the megasynthase enzymes encoded by five different genes (*TylG*I-*TylG*V). Four different thioester-CoAs are selected by AT domains of each module. Each selected extender units are modified to a certain degree by respective reducing enzymes, except the module 4 in which KR domain is null functional. In the end, the PKS product is off-loaded by the action of TE domain present in the last module 7, encoded by *TylGV* gene. Once the PKS product is released, the biosynthesis is further extended by post-PKS tailoring enzymes such as cytochrome P450 monooxygenase (CYP450), glycosyltransferases (GTs) and methyltransferases (MTs) [26].

**Fig. 3:**
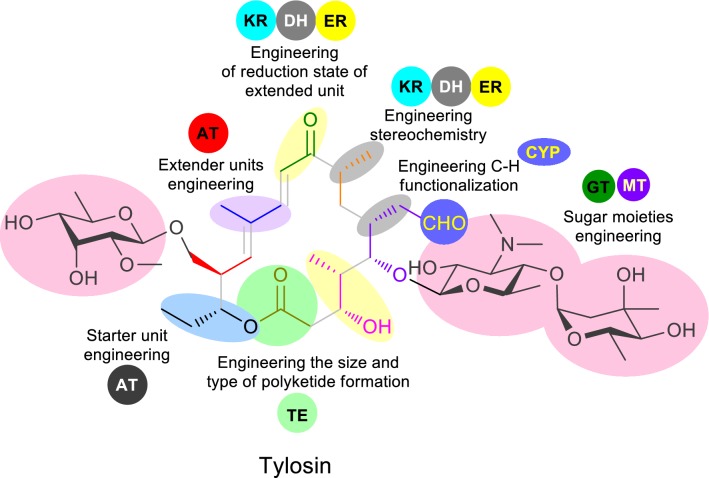
Structure of tylosin showing possible modification/engineering sites for engineering/diversification of tylosin and related molecules

Despite the numerous reports on the “canonical” organization and co-linearity in most of the type I PKSs, non-canonical examples of type I PKS are popular [27]. The skipping over of the extraneous functional domains or modules leading to the formation of unusual intermediates or final compounds have been reported [28, 29]. Similarly *trans*-acting domains have been uncovered from various polyketide biosynthesis. These *trans*-enzymes are usually physically separate from the usual modifying domains in the modular PKSs, but they act at specific points during the processing of the nascent acyl chains. One of the prominent examples is *trans*-AT or AT-less biosynthesis of polyketides whereas AT activity is provided at each elongation step in *trans* by free-standing AT usually encoded in the biosynthetic gene clusters [30]. *Trans*-ER has been characterized from lovastatin polyketide biosynthesis [31] and switchable ER domain has been reported in azalomycin F biosynthesis [32]. Similarly, some *trans*-acting thioesterases (TE type II) are responsible for editing function by hydrolytic removal of aberrant residues blocking the megasynthase, participation in substrate selection, intermediate, and product release [33]. Similarly broadly selective acyltransferase has been characterized from the polyketide synthase of splenocin [34]. The detailed information on extended unit promiscuity and orthogonal protein interaction of ACP and trans-AT has been also availed [35, 36]. So, these type of new information are not only providing novel insight on the biosynthetic mechanism of polyketides but also providing opportunities for plausible bio-engineering of these macrolides. Basically, the diversification of macrolides can be tuned at four major steps throughout biosynthesis through (1) choice of the building block and chain length, (2) reduction and stereochemical arrangement of β-keto intermediates, (3) rearrangements and secondary cyclizations and (4) post-PKS tailoring [37]. Recent information on the protein structure of the enzymes and advanced genetic manipulation techniques provides enormous opportunity for fine tuning the post-PKS steps to generate novel or structurally diversified macrolides [38, 39]. Figure 3 depicts the plausible modification centers in macrolide that can be altered by utilizing different engineering aspects in the actinobacteria either by rational modification on PKS or post-PKS steps.

## Precursor-directed biogenesis of novel antibiotics

The prolific generation of mutant bugs to the many pre-existing drugs has caused pitfall in efficiency of enhancement approach as a panacea for addressing the issues of drug resistance. Nevertheless, precursor directed biogenesis can still substantiate as a promising technique by providing a fertile platform for structurally diversifying the antibiotics, whereas the altered natural product precursors are subjected to the microorganisms and subsequently incorporated to reconstitute the product of interest [40]. The alternative precursor-directed biosynthesis can be utilized as an approach where the chemical and biological inputs are assembled for generation of novel natural products. For example, an orally active β-lactam antibiotic, penicillin V (phenoxymethylpenicillin), was produced by adding phenoxyacetic acid to fermentations of *Penicillium chrysogenum* [41].

But the major drawbacks of this approach can be inherent competition between alternative precursors and natural precursors, rendering the yield of novel derivatives to be low. However, this shortcoming can be overruled by blocking the synthesis of natural precursors, either by mutating key genes in the respective bio-synthetic pathway or by adding specific inhibitors of biosynthetic enzymes [42]. This approach in which the biosynthetic pathway is coupled with the feeding of synthetic starter precursors, where the precursors are reorganized in the biosynthetic pathway to remold the final compound can be otherwise be termed “chemosynthetic biogenesis”. Hence utilizing the benefits of this approach, various novel analogs of erythromycin and other polyketides are generated [43–47].

**Fig. 4:**
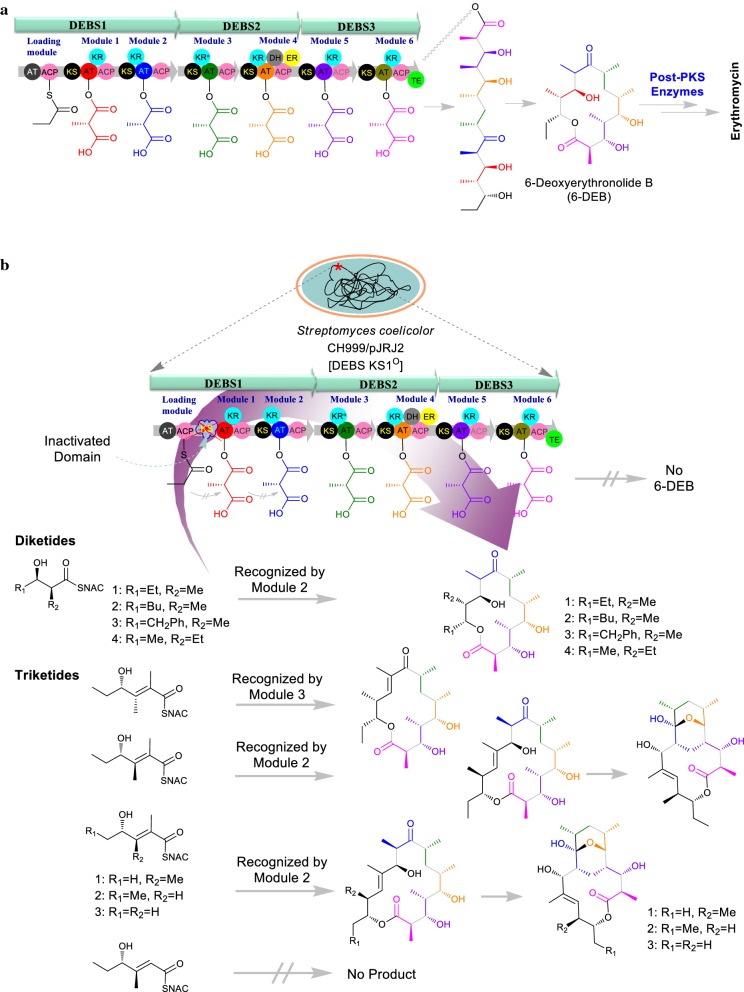
**a** Biosynthesis pathway assembly of erythromycin. **b** Biosynthesis of diverse erythromycin derivatives using *Streptomyces coelicolor* deficient in KS1^0^. Different activated synthetic diketides and triketides were supplemented to the culture

Fundamentally for generating diversities of analogs of erythromycin, a designed mutant of a modular PKS by inactivation were generated, that lacked early-stage enzyme activities. DEBS (KS1^0^), a mutant of DEBS that was inactivated by site-directed mutagenesis of the β-ketoacyl-ACP synthase domain of module 1 (KS1) was used for precursor mediated structural diversification strategy [42]. The native KS1 domain catalyzes the first condensation step of 6-deoxyerythronolide B (6-dEB) biosynthesis (Fig. 4a) but the DEBS (KS1^0^) mutant is incapable of carrying out the first round of polyketide chain elongation from the propionyl-CoA and methylmalonyl-CoA causing incapability for the formation of the macrolide 6-dEB. In turn, by the introduction of synthetic diketide and triketide such as the *N*-acetyl cysteamine (SNAC) thioester, which are analogs of the natural diketide intermediate to *Streptomyces coelicolor* CH999/pJRJ2, an engineered strain harboring DEBS (KS1^0^), various novel analogs of erythromycin were generated (Fig. 4b) [48].

## Mutasynthesis

The precursor directed biogenesis has been utilized and still remains as a promising strategy for structurally diversifying and generating versatile chemical identities with superior properties or activities. But, the major drawback clinging to these approaches, most important being that mixture of natural and nonnatural products with similar physical properties are often produced, leading to complex downstream purification procedures; high concentrations of synthetic precursor are often required to compete with the preferred natural precursor and; a limited range of intermediates are efficiently incorporated into the final product. So, the precursor-directed biosynthesis has been complemented with mutasynthesis approach whereas the naturally occurring precursor pathways are inactivated by mutation to remove the competition with natural precursors.

*Streptomyces avermitilis* mutant (*S. avermitilis Δbkd*) was generated wherein the enzymes, branched chain fatty acid dehydrogenase complex Bkd required for generating the precursors 2-methylbutyryl-CoA and isobutyryl-CoA were inactivated. Different precursor analogs were fed to the mutant and a cyclohexyl-containing avermectin derivative (later named doramectin) was generated with increased antiparasitic activity [49]. Similarly, the deletion of 5-chlorodeoxyadenosine (a precursor for chloroethylmalonyl-CoA) biosynthetic genes in *Salinispora tropica* [50] and exogenous supplementation of 5-fluorodeoxyadenosine resulted in the generation of fluorosalinosporamide.

## Combinatorial biosynthesis for diversification of antibiotics

“Combinatorial biosynthesis” is one of the promising strategies for the genesis of novel analogs of predominant antibiotics or modifying their structural aspects for better pharmacological properties. This approach intervenes with the strategies for the genetic engineering of natural product biosynthesis to obtain new molecules, including the use of genetics in medicinal chemistry [51]. It has been an important approach to generate chemical diversity by precise genetic manipulations and thus implies with the possibility of generation of large libraries of complex compounds to feed a modern high-throughput screening operation [51, 52]. Thus, combinatorial biosynthesis is a recent addition to the metabolic engineering toolbox by which genes responsible for individual metabolic reactions from different organisms are combined to generate the metabolic pathways to biosynthesize the desired products [53]. Fundamentally, it can be distinguished from mutasynthesis approach on the bases that a mutasynthesis is an approach where there is inactivation of some key functional genes, thus perturbing the biosynthesis pathway to new products by supplementation of feasible precursors or chemical entities; whereas combinatorial biosynthesis rely on additional gene functions by heterologous expression of functionally similar or diverse genes so that the pathway is tuned for crafting novel analogs. It has been widely applied to achieve new derivatives related to polyketides, coumarins, indolocarbazoles and other types of antibiotics [53–55].

Among the compounds that have been developed into medicines, including the antimicrobials and antibiotics, the polyketides occupy significant part. The polyketides are produced as a diverse array of products manifested by the bacterial megaenzyme polyketide synthases (PKSs). The distinct modularity in the genetic architecture of these PKSs provides sufficient ground for expecting feasibility for engineering the enzymes to produce novel drug candidates, by ‘combinatorial biosynthesis’ [56].

The supplementation of the shikimate-derived cyclohexyl-CoA biosynthetic pathway into *S. avermitilis* Δ*bkd* enabled the production of doramectin without cyclohexanoic acid supplementation [57]. Similarly, supplementation of diverse carboxylic acid starter unit to spinosyn PKS containing loading modules of avermectin or erythromycin could generate diverse spinosyn analogs [58]. Similarly, multiple bioactive macrolides were generated by hybrid modular PKS from pikromycin gene cluster, erythromycin gene cluster, and tylosin gene cluster, whereas rigorous interchange of modules is employed between the PKSs [59]. This work demonstrated the unique capacity of combinatorial biosynthesis for accelerating the creation of novel biologically active natural products.

**Fig. 5:**
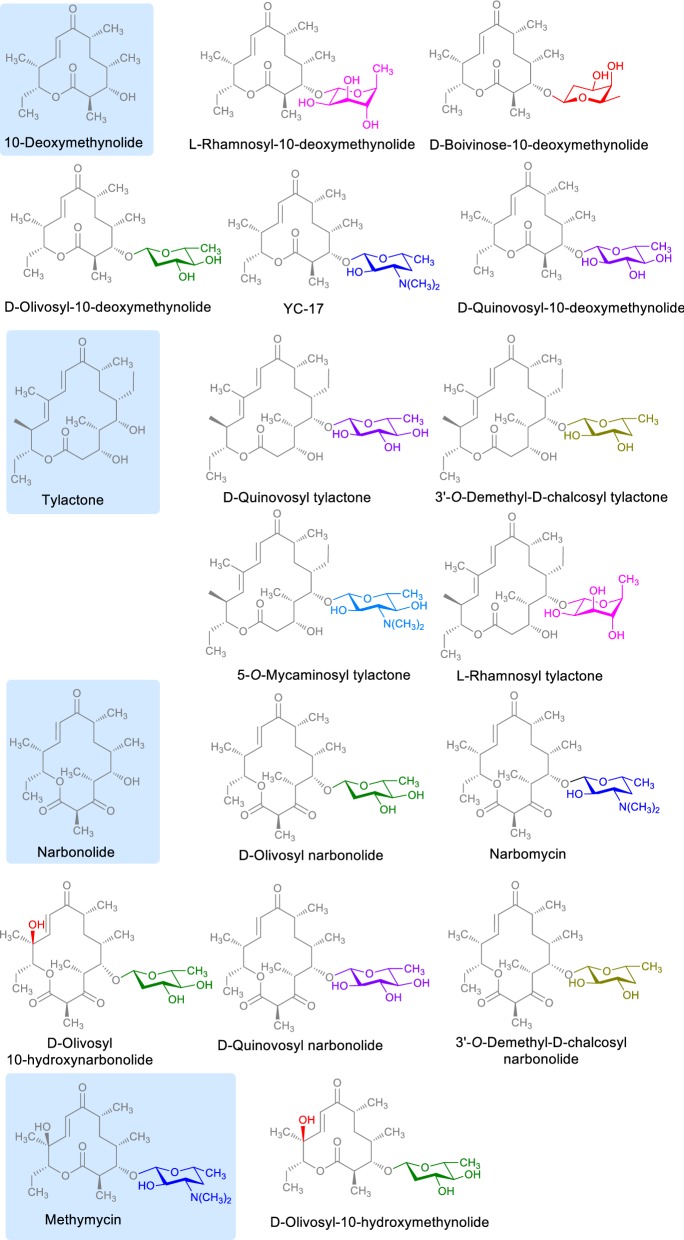
Structures of different sugars conjugated macrolides produced using TDP-sugars biosynthesis pathway engineered *Streptomyces* recombinant strains

By utilizing *S. venezuelae* based combinatorial biosynthesis machinery, many novel analogs of various secondary metabolites with diverse structure and diverse activities have been generated [60–64]. *S. venezuelae* ATCC 15439 was engineered for deletion of genes responsible for biosynthesis and attachment of TDP-4-keto-6-deoxy-d-glucose and the strain was designated as *S. venezuelae* YJ003. The sugar gene cassettes encoding for deoxysugar biosynthesis and glycosylation were expressed in the engineered strain to generate olivosyl and quinovosyl derivative of various macrolides [60]. *S. venezuelae* was genetically engineered for deletion of the entire biosynthetic gene cluster encoding the pikromycin PKS and desosamine biosynthetic enzyme [61]. After crafting the amenable host for combinatorial biosynthesis, it was used multifarious for generation of different analogs of targeted polyketides. The engineered deoxysugar biosynthetic pathways for biosynthesis of thymidine diphosphate (TDP)-d-quinovose or TDP-d-olivose along with substrate flexible glycosyltransferase–auxiliary protein pair DesVII/DesVIII from *S. venezuelae* were expressed in the mutant strain to which 12-, 14-, and 16-membered ring macrolactones i.e. 10-deoxymethynolide, narbonolide, and tylactone, respectively were feed to generate corresponding quinovose- and olivose-glycosylated macrolides. The conversion of 12-, 14-, and 16-membered ring macrolactones including 10-deoxymethylnolide, narbonolide, and tylactone were achieved in engineered *S. venezuelae* strain with deoxysugar biosynthetic pathways (TDP-d-quinovose or TDP-d-olivose) together with glycosyltransferase-auxiliary protein (DesVII/DesVIII) and produce their glycosylated scaffolds as quinovosyl and olivosyl macrolactones. The synthesized compounds were YC-17 and narbomycin. Similarly, while replacing the DesVII/DesVIII by substrate-flexible glycosyltransferase TylMII coupled with partner protein TylMIII derived from *S. fradiae*, a mycaminosyl derivative of tylactone (5-*O*-mycaminosyl tylactone) was produced [61]. Similarly, expression of complete biosynthetic pathways for the biosynthesis of TDP-3-demethyl-d-chalcose or TDP l-rhamnose together with the glycosyltransferase-auxiliary protein pair DesVII/DesVIII along with subsequent feeding of 16-membered ring macrolactone tylactone was fed to this engineered host, which in turn successfully produced 3-*O*-demethyl-d-chalcosyl, l-rhamnosyl, and d-quinovosyl derivatives [62]. Similarly, using *S. venezuelae* YJ003 and expression of complete biosynthetic pathways for the biosynthesis of TDP-3-dimethyl-d-chalcose or TDP-l-rhamnose together with DesVII/DesVIII, novel narbomycin derivative decorated with l-rhamnose or 3-*O*-demethyl-d-chalcose were generated. These novel analogs exhibited greater antibacterial activity than narbomycin and the clinically relevant erythromycin [62]. In another instance, for another aglycone YC-17, the native d-desosamine was replaced by d-quinovose, l-olivose, l-rhamnose, and d-boivinose to generate YC-17 glycoside analogs as d-quinovosyl-10-deoxymethynolide, l-olivosyl-10-deoxymethynolide, l-rhamnosyl-10-deoxymethynolide, and d-boivinosyl-10-deoxymethynolide respectively by expression of gene cassette responsible for biosynthesis of respective deoxysugars (Fig. 5). The assessment of biological activity indicated that l-rhamnosyl-10-deoxymethynolide exhibited better activities against clinically isolated erythromycin-resistant pathogenic strains, as well as erythromycin-susceptible strains relative to YC-17 and its other analogs [64]. These all studies indicate that the combinatorial biosynthesis mediated structural diversification can be one of the efficient techniques for modifying the structure and thus rendering enhancement in activity mediated by the structure–activity relationship as par distinct structural scaffold contributing for a particular range of activity.

**Fig. 6:**
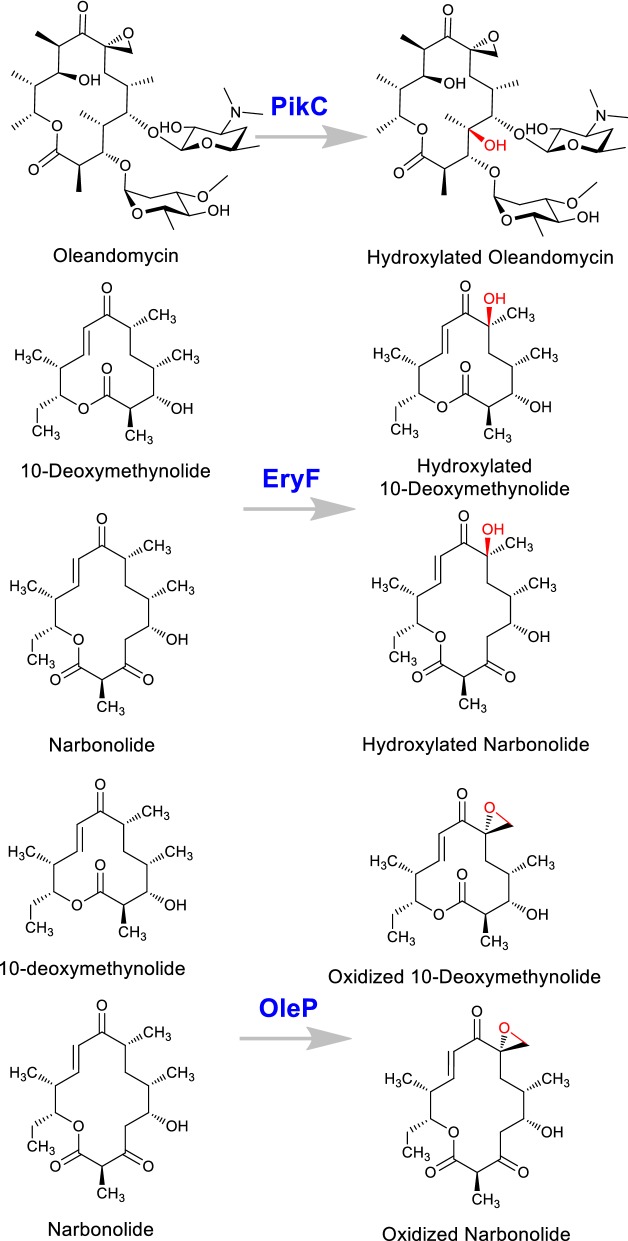
Generation of different macrolactones using selected enzymes such as PikC, EryF and OleP

The large multifunctional PKS enzymes synthesize macrolides [65]; which undergoes multiple modifications by post-PKS enzymes mediated reactions such as oxidation, methylation, glycosylation, hydroxylation and many more giving rise to chemically distinct structures. The functional and structural diversity of these compounds is controlled by these post-modifications and most often these modifications are critical for biological activities [38]. The hydroxylation or other modifications contributed by cytochrome P450 monooxygenases are the key steps leading to structural diversity and biological activities to macrolide antibiotics [66]. Hence, by utilizing the substrate flexible cytochromes for combinatorial biosynthesis novel analogs were generated. An engineered *S. venezuelae* HK954 mutant strain (with deletion of the last module of pikromycin PKS (*pikAIV*) but intact substrate flexible cytochrome P450, pikC) was used for generating novel hydroxylated analogs of oleandomycin [67]. Similarly, *S. venezuelae* mutant strain YJ003 blocked in desosamine biosynthesis pathway was used for the expression of substrate flexible cytochrome P450s, EryF from erythromycin gene cluster and OleP from oleandomycin gene cluster to generate novel analogs of 12 and 14 membered macrolactones inherently produced by the host strain (Fig. 6) [67].

## Metabolic engineering approaches in actinomycetes for macrolide biosynthesis

The major constraint for biosynthesis of macrolides in the producer-host has been abundant availability of precursors and cofactors, the lower expression level of biosynthetic genes and regulation of the biosynthetic gene/genes. Generally, the bottlenecks in biosynthetic pathways restrict the substantial production of the natural product in desired titers. This is significantly associated with the limitations of the flux of key precursors from primary metabolism to secondary metabolic pathways [68, 69]. Generally, the precursors/cofactors are derived from primary metabolism including glycolysis, pentose phosphate pathway, tricarboxylic acid cycle and amino acid/nucleic acid metabolism [70]. Thus these types of limitations can be overcomed by amplifying the gene or genes that encode enzymes associated with such bottlenecks resulting in increased enzyme levels to diminish the bottleneck effects and hence, improved titers can be achieved [71, 72]. Fundamentally, regulation and correlation of precursor supply for improving natural product amounts are focused on carbohydrate metabolism, fatty acid precursors, and intracellular cofactor supplies [73, 74]. There are ample of examples illustrating the genetic circuit guided pathway engineering approaches for enhancing the secondary metabolites of importance [75], such as heterologous overexpression of the S -adenosyl-L -methionine (SAM) synthetase *metK*, improved production of different antibiotics, such as actinorhodin, avermectin, and pikromycin [76, 77]. For example, the engineering of methylmalonyl-CoA biogenesis pathway by generating MCM gene, methylmalonyl-CoA mutase (*mutB*) knock out mutant of *S. erythraea*, there was an enhanced level of erythromycin in the carbohydrate-based medium. However, there was an elevated level of erythromycin in *S. erythraea* recombinant containing MCM operon (*meaA*, *mutB*, *meaB*, *mutR*) in the oil-based medium [78]. Similarly by supplementation of cheap primary sources which subsequently contribute for specific fatty acid precursors viz. acetyl-CoA, malonyl-CoA, methyl malonyl-CoA and ethyl malonyl-CoA along with precursor redirecting enzyme complexes such as acetyl CoA carboxylase, and propionyl CoA carboxylase different antibiotics such as nargenicin A1, pikromycin, and erythromycin were elevated to significant level [79–82]. Hence, metabolic engineering combined with redirection of specific precursors can be a rational approach for enhancing the secondary metabolites of importance. Similarly, such approaches for enriching the acyl-CoA precursors have been used for an elevated level of diverse macrolides (Table 1). Recently, various engineering strategies for directing the catabolism of branched-chain amino acids (BCAA) into various acyl-CoA compounds has extended the opportunities for metabolic engineering of acyl-CoA pathways and yield improvement of macrolides [83].

**Table 1:** Metabolic engineering approaches used for enhancing the production of diverse macrolides

Compound	Strain	Approach	References
Native host			
Epothilone	*Sorangium cellulosum*	Expression of propionyl-CoA synthetase, which converts propionate into propionyl-CoA, the precursor of methylmalonyl-CoA	[148]
Nargenicin A1	*Nocardia* sp. CS682	Over-expression of ACCase complex for enhancement of production	[82]
Natamycin	*S. chattanoogensis* L10	Overexpression of the endogenous PPTase SchPPT for enhancement of production	[149]
Tylosin	*S. fradiae*	Different approaches for enhancement of production titer: SARP regulators *tylS* and *tylR* are overexpressed Inactivation of a transcriptional repressor (*tylQ*) Diruption of *tylP* encoding a γ-butyrolactone receptor	[150–152]
Spiramycin	*S. ambofaciens*	Overexpression of pathway-specific regulator *srm22* or *srm40* increased spiramycin production	[153]
Milbemycin	*S. bingchenggensis*	Enhancement by overexpression of pathway-specific regulator *milR*	[154]
Pikromycin	*S. venezuelae*	Enhancement of production by overexpressing genes of branched chain amino acids catabolism	[83]
Stambomycins	* S. ambofaciens*	Overexpression of pathway-specific regulator *samR0484 triggers production of cryptic BGC*	[155]
Heterologous hosts			
FK 506	*S. clavuligerus* CKD1119	Expression of *mutB* from *S. erythraea*	[156]
Methoxymalonate-platenolide analog	*S. fradiae*	Introducing the biosynthetic pathway for methoxycarbonyl-ACP	[112]
Midecamycin	*S. fradiae*	Enhancement of production by introducing the biosynthetic pathway for methoxylmalonyl-ACP from the FK520 producer *S. hygroscopicus*	[112]
Pikromycin	*S. venezuelae*	Enhancement of production by expression of two global regulators, metK1-sp and afsR-sp, from *Streptomyces peucetius*	[157]
Tylosin	*S. venezuelae*	Overexpression of *pikD*, a pathway-specific regulator from pikromycin biosynthetic pathway	[158]

Similarly, the expression of regulatory genes has been promising strategies to enhance the production titer or activation of novel macrolides in diverse actinomycetes. Generally, the introduction of synthetic or natural promoter has been effective for triggering either overproduction or activation of silent/cryptic gene clusters with low or no expression [84–89]. The metabolic engineering approaches utilizing overexpression/deletion of various global regulators or pathway-specific positive regulators are utilized for either enhancing the production yield or obtaining novel macrolides from native hosts/heterologous hosts (Table 1).

The availability of the crystal structure of complete module of PKS [16, 90] or its constituent domains as ACP [91], dehydratase [92], thioesterase [93, 94] has provided a better understanding on the mechanism of macrolactone biosynthesis. In case of polyketide biosynthesis, the AT domain is crucial for controlling the recognition of the extender unit. Thus, there is a possibility of changing the building block specificity and the basic backbone of the polyketide by altering the AT domain. Such valuable information has been utilized for rational engineering of macrolactones. The site-directed mutagenesis of the domains or intact domain exchange to change their substrate specificities has been widely used for generating diverse analogs of the macrolides, which have been reviewed elsewhere [13]. The exchange of AT domain by domain swapping can cause impaired protein folding [95]. However, Yuzawa et al. identified the highly conserved boundaries and exhibited the feasibility of AT domain replacement in macrolides [96]. But the direct engineering of innate AT domain can be a more reliable alternative for varying the substrate ranges [97]. For example, the site-directed mutagenesis of AT domain and feeding of 2-propagylmalonyl-SNAC to *S. erythraea* (containing AT6 of DEBS mutated with Val295 to Ala) to generate 2-propargyl erythromycin [98]. The mutation is not only capable of extending the substrate specificity to natural extender units but also alter the specificity non-natural extender units. For example, the selected mutation at Tyr189 in DEBS3 AT6 domain resulted in the dramatic changes in product distribution by accepting diverse non-natural extender units [99].

Similarly, novel derivatives of NPP have been generated by site-directed mutagenesis in enoyl reductase domain in module 5 of the NPP A1 polyketide synthase NppC. The compound exhibited comparable antifungal activity against *Candida albicans* with lesser toxicity than antifungal, amphotericin B [100]. However, such kind of domain modification may create inactive proteins or change the chemistry of inter-domain interactions. So entire domain exchange has been a superior option and several analogs of erythromycin have been generated by replacing methylmalonyl-specific acyltransferase (AT) domains of the 6-deoxyerythronolide B synthase (DEBS) with malonyl-, ethylmalonyl-, or methoxymalonyl-specific domains [101].

**Fig. 7:**
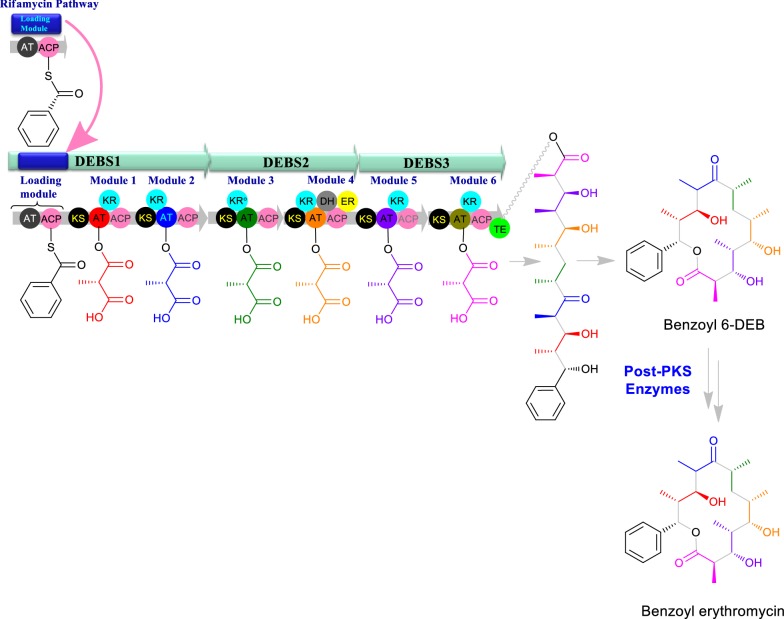
Biosynthesis of benzyoyl erythromycin by replacing loading module of erythromycin from loading module of rifamycin biosynthesis pathway

Similarly, metabolic engineering approach has been performed for complete exchange of the module. For example, the loading module from the rifamycin biosynthetic pathway as a substitution for the original loading domain was incorporated in the PKS so that the altered PKS was amenable for accepting benzoate as starter unit instead of the propionyl-CoA used in native. By this strategy, a novel benzyl-erythromycin analog was generated and by utilizing further precursor flux enhancement and pathway engineering approaches the titer of the novel derivative was substantially increased (Fig. 7). The novel derivative was capable to show comparable activity to the parent compound whereas showing pronounced efficacy against the erythromycin resistant pathogens [102]. Similarly, the domain swapping of pikromycin thioesterase to linear polyketide tautomycetin (TMC) was able to generate the cyclized form of macrolactones [103].

In general, most of the actinomycetes strain is capable of generating profuse secondary metabolites with different structures or activities. Most often these chemical entities share common scaffold and thus similar precursor régimes. Thus, the precise and efficient manipulation of modular PKSs is often hindered by biological constraints posed by organisms (principally actinomycetes) due to their intricate biological pathways and complex distribution of precursor flux for the constitution of different secondary metabolites. To address this constraint, an heterologous expression host as *Escherichia coli* was engineered with the introduction of the three DEBS genes from *S. erythraea*, the *sfp* phosphopantetheinyl transferase gene from *Bacillus subtilis*, and the genes encoding a heterodimeric propionyl-CoA carboxylase from *S. coelicolor* [104]. This engineered *E. coli* BAP1 strain was utilized for fortifying generation of novel analogs of erythromycin (Fig. 7), as described previously. Further, the production was fine-tuned by reducing the total number of plasmids [105] and utilizing the BAC vector [106]. The metabolic engineering approach by eliminating competitive native pathway for nucleotide-activated sugar biosynthesis pathways (*vioA/vioB/wzx*, *wecD/wecE*, and *rmID*) substantially increased the production titer [106]. The metabolic engineering approach was employed for expanding the formation of novel erythromycin analogs by altering the tailoring enzymes involved in sugar biosynthesis. Among them few of the novel analogs exhibited promising activity against the erythromycin-resistant strain of *B. subtilis*, which provided a rationale of this diversification strategy for increasing the therapeutic potential of erythromycin [107].

Thus heterologous expression has been an efficient approach of metabolic engineering where a single gene, or a set of genes, or entire biosynthetic pathway genes are introduced in the microbial host to identify and engineer the corresponding natural products [108]. When if the native producer strains are not genetically tractable or not amenable to metabolic engineering approaches, the heterologous hosts provide an efficient approach for gaining access to secondary metabolites encoded by the particular BGCs. In addition, the generation of metabolic engineering by removing the competing biosynthetic pathways to generate clean hosts provides a suitable platform for isolation, characterization, and production of various macrolides [109, 110]. The generation of cluster free clean hosts provides new avenues for redirecting the flow of precursor pathways directly to the target secondary metabolite heterologously expressed [111]. Diverse macrolide biosynthesized through heterologous hosts are presented in Table 2. In some cases, the heterologous expression of the BGCs needs to be tuned by expression of the regulatory genes. For example, the heterologous expression of a tylosin gene cluster in *S. venezuelae* was enhanced by expression of *pikD*, a positive regulator from pikromycin biosynthetic pathway of *S. venezuelae*. In other cases, the tuning of heterologous expression by an engineered precursor pathway can enhance the production titer or generate novel derivatives in heterologous expression systems [112]. The introduction of the biosynthetic pathway for methoxymalonyl-ACP into an *S. fradiae* strain heterologously expressing the midecamycin pathway led to enhancement of midecamycin and generation of a novel compound as an analog of methoxymalonate-platenolide [112].

**Table 2:** Diverse macrolide biosynthesized through heterologous hosts

Compound	Producer-host	Heterologous host	References
Avermectin	*S. avermitilis*	*S. lividans*	[159]
Chalcomycin	*S. bikiniensis*	*S. fradiae*	[160]
Epothilone	*Sorangium cellulosum*	*S. coelicolor*, *M. xanthus*, *E. coli*, *S. venezuelae*; *Myxococcus xanthus Burkholderiales* strain DSM 7029	[117, 161–166]
Erythromycin	*Saccharopolyspora erythraea*	*S. coelicolor*, *E. coli*	[167, 168]
Megalomycin	*Micromonospora megalomicea*	*Saccharopolyspora erythraea, S. lividans*	[169]
Spinosyn	*Saccharopolyspora spinosa*	*S. albus*	[118]
Tylosin	*S. fradiae*	*S. venezuelae*	[170]
Midecamycin	*S. hygroscopicus*	*S. fradiae*	[112]
Pikromycin	*S. venezuelae*	*S. coelicolor*, *S. lividans*	[171, 172]
Oleandomycin	*S. antibiticus*	*S. coelicolor,*	[171]
Versipelostatin	*S. versipellis* 4083	*S. albus*	[173]
Galbonolide B	*Streptomyces* sp. LZ35	*S. coelicolor*	[174]
FK506	*S. tsukubaensis*	*S. coelicolor*	[175]
Streptoseomycin	*S. seoulensis*	*S. chartreusis*	[176]
Nemadectin	*S. cyaneogriseus*	*S. avermitilis*	[110]
bafilomycin B1	*Kitasatospora setae* KM-6054	*S. avermitilis*	[110]
Quinolidomicin	*Micromonospora* sp. JY16	*S. lividans*	[173]

**Fig. 8:**
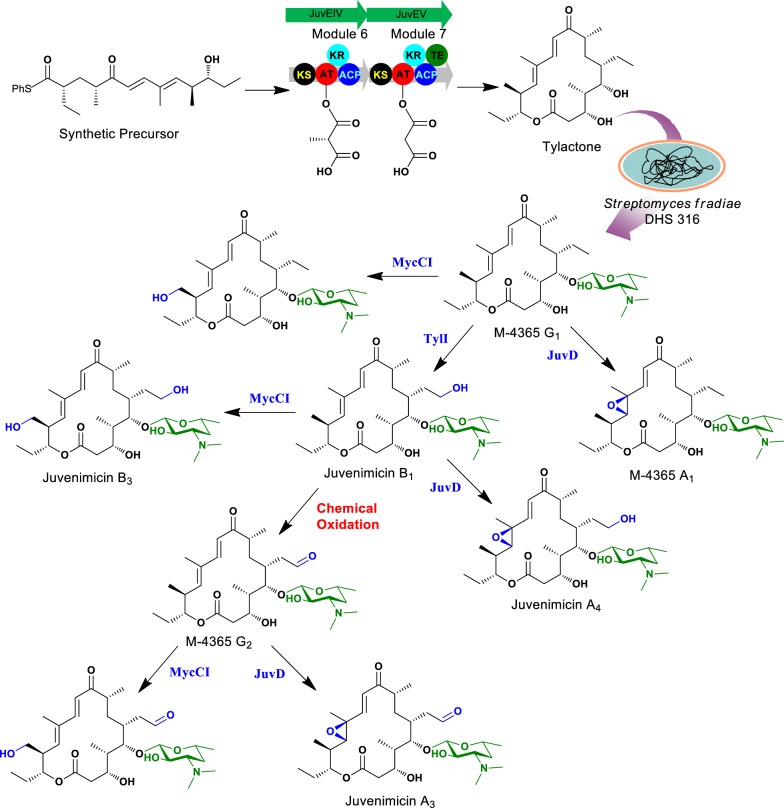
Chemoenzymatic and biocatalytic synthesis of structurally diverse tylactone-based macrolides antibiotics

Recently, the biocatalytic/chemo-biocatalytic approach employing a heterologous expression of the partial PKS and substantial modification has been employed as an effective approach for generating novel macrolides [113]. For example, the Pik pentaketide precursor was supplemented to the expressed protein of PikAIII-TE or PIKAIII-PikAV to generate the macrolactones, 10-deoxymethynolide, and narbonolide. Further biotransformation with engineered *S. venezuelae* could generate diverse derivatives related to pikromycin and methymycin. Similarly, activated synthetic hexaketide was further extended with a methyl malonyl and malonyl units followed by lactonization to form tylactone aglycon by in vitro catalysis using tylosin biosynthesis complementary modules (module 6 and 7 encoded by *JuvEIV* and *JuvEV* genes) from juvenimicin BGC. The tylactone was further biotransformed to M-4365 G_1_ using *S. fradiae* DHS 316 which is deficient of tylactone BGC. This intermediate was modified into ten different macrolide derivatives using CYP450 and chemical oxidation methods [114, 115] (Fig. 8). This approach advanced the use of type I PKS enzymes in vitro for generation of novel antibiotics.

However, this method suffers from the limitations of scalability whereas the starting materials need to be supplied in sufficient amounts for sustainable production. In addition, there is a requirement of strict control in unwanted reactive centers but enough reaction in desired reaction centers, which makes these synthesis processes tedious and expensive. Thus, biological engineering of macrolides by using sustainable metabolism in the microbial hosts may always remain superior. Recently, synthetic biology approach which utilizes the artificial synthesis of genetic parts in revolutionizing such biological engineering aspects. For example, the promoter engineering by utilizing the regulatory sequences in the promoters can be an effective approach for tuning the production titer of macrolides [116]. The biosynthesis of epothilone was successfully accomplished by modular construction of artificial macrolide pathway [117]. The availability of detailed knowledge of diverse –omics related information can be successfully employed for fine tuning the heterologous expression of such macrolide compounds [118].

## Microbial biotransformation for modification of antibiotics

Besides the precise genetic engineering approaches, microbial biotransformation strategies have attracted considerable interest for the post-modification of secondary metabolites in terms of their structural and functional characteristics [119–121]. Any changes in parental compounds in a later stage can refer to as post-modification in terms of structure and biotransformation is one of the well-studied and precise approaches to achieve such modifications with an optimal regio- and enantio-selectivity [122, 123]. However, the major modification includes cofactor-dependent hydroxylation/oxidation, dehydrogenation, methylation, glycosylation (*O*/*C*/*N*-glycosylation, deglycosylation), epoxidation, cyclization etc. [124–127], whereas the intracellular cofactors/enzymes present in the biotransformation host is responsible for desired modification of the supplemented substrate. Such whole-cell biotransformation can be achieved using various microbial species (*Streptomyces*, *Myxobacterium*, *Bacillus*, *Corynebacterium*, *Pseudomonas*, etc.) as a host.

**Fig. 9:**
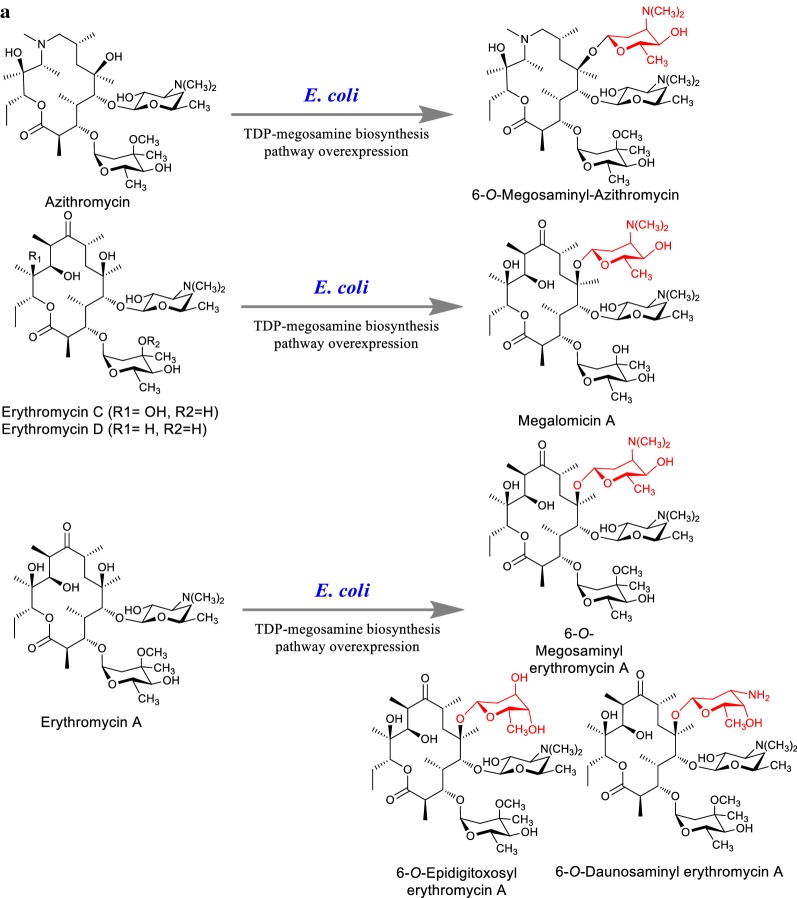

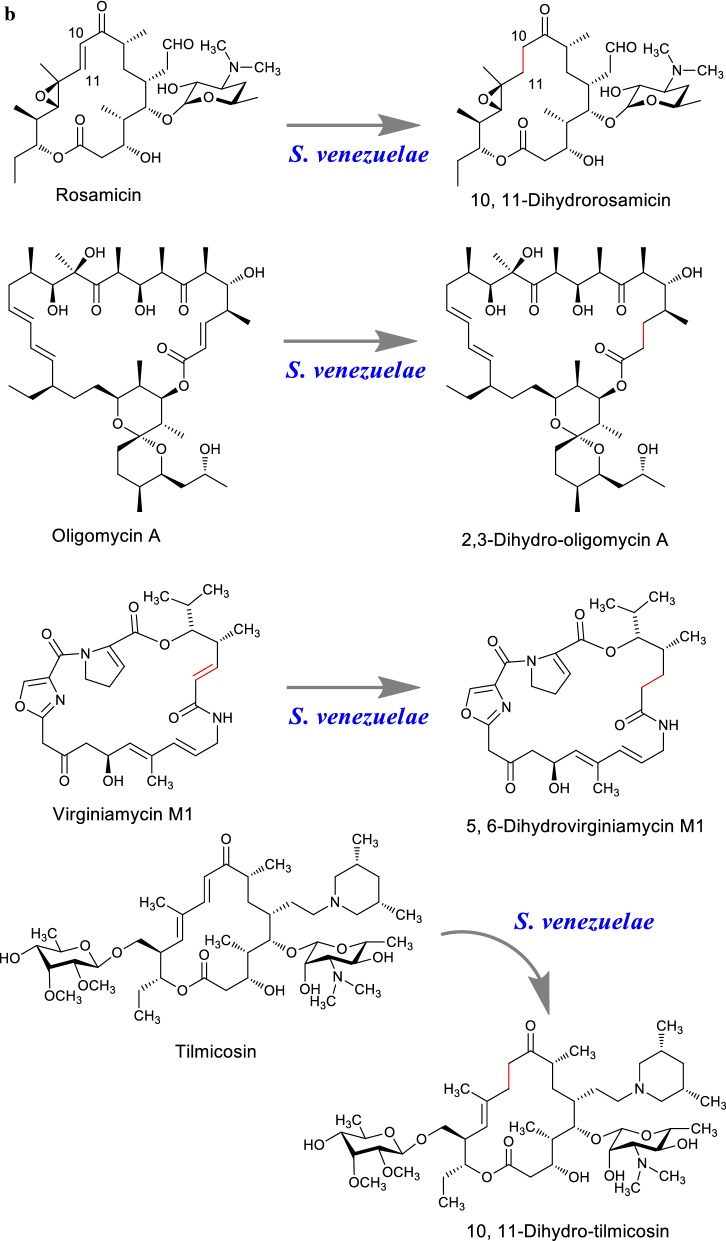
**a** Biosynthesis of glycosylated macrolides from different TDP-sugars (TDP-L-mycarose, TDP-desosamine, TDP-megosamine) biosynthesis pathway overexpressed recombinant *E. coli* strains supplemented with corresponding macrolactone/macrolides. **b** Biotransformation of macrolides using *Streptomyces venezuelae* strain

A series of erythromycin D analogs conjugated with different sugar scaffolds were produced feeding 6-deoxyerythronolide B or 3-α-mycarosyl-erythronolide B in *E. coli* overexpressing the specific TDP-L-mycarose and TDP-desosamine sugar biosynthetic pathway [128]. Same biotransformation set-up was used for modification of erythromycin C, 3-α-mycarosyl-erythronolide B, azithromycin, and erythromycin D to modify them into megosamine conjugated products using reconstitutive TDP-l-megosamine biosynthetic pathway in *E. coli* [129, 130] (Fig. 9a). This experiment also suggested the possible routes for the production of diverse therapeutically important (antiparasitic, antiviral and antibacterial) compound megalomicin A including 12-deoxynucleoside triphosphate megalomicin A [130]. Importantly, sugar appendages present in macrolide antibiotics governs their biological properties and changes in them have a substantial effect [131]. So, deoxy sugar biosynthetic pathways are usually focused in terms of antibiotic modifications. 6-*O*-megosaminyl-erythromycin A, 6-*O*-megosaminyl-azithromycin, 6-*O*-epidigitoxosyl-erythromycin A and 6-*O*-daunosaminyl-erythromycin A were produced through the whole cell biotransformation. Their evaluation against several clinical isolates; standard and drug-resistant strains of human malarial parasites (*Plasmodium falciparum*) and liver stages of the rodent malaria parasite (*Plasmodium berghei*) were found more effective than parental compound [130].

Rosamicin, macrolide antibiotics isolated from the culture of soil bacterium was bio-transformed into 10, 11-dihydrorosamicin using *S. venezuelae* which showed enhanced in vitro antibacterial activity against MRSA [132]. Likewise, natural oligomycin A and semi-synthetic tilmicosin are considered and bio-hydrogenated using *S. venezuelae* and structurally modify into 2, 3-dihydro-oligomycin A and 10, 11-dihydro-tilmicosin (Fig. 9b). This changes in natural scaffold oligomycin and tilmicosin brought increased activity against *S. cerevisiae* and *Bacillus subtilis* respectively [133]. Similarly, macrocyclic lactone antibiotic streptogramin A namely 5, 6-dihydrovirginiamycin M1 was created by feeding virginiamycin M1 into a culture of recombinant *S. venezuelae* through the mechanism of bio-dehydrogenation catalysis [134]. The generated analog showed enhanced anti-MRSA activity compared to the parent compound.

## Conclusions and future perspectives

Actinobacteria have been already established as the promising platform for the production of macrolides and their novel derivatives. Moreover, the recent advances in various “–omics” provides new paths for uncovering the genomic information and their expression levels. Moreover, the availability of various bioinformatics tools for classifying the genome to BGCs and analysis of the products by versatile metabolome analyzing tools helps in connecting the genome to a particular molecule, which is also otherwise called as “genome mining” approach [135–139]. In addition, the current success of enzyme/host engineering has narrowed down the gap between the understanding of PKS biosynthetic logic and its propensity for PKS diversification. The advances in genome sequencing, protein crystallography, and gene-synthesis system have made it feasible for designing, building and testing chimeric PKSs. The advancement in the analytical methods by mass spectrometry and molecular networking have increased our capacity for detecting the products [140]. The feasibility of the optimization of production hosts either native or heterologous by computational and systems biological tools [141] provides an effective or alternative route for fluxing the precursors toward the biosynthesis from such chimeric PKS. Various genome engineering techniques such as Multiplex Automated Genome Engineering (MAGE) [142, 143] and Clustered Regularly Interspaced Short Palindromic Repeats (CRISPRs)/CRISPR associated enzyme (Cas) [144, 145] provides ample of opportunities for precise metabolic engineering of the PKS and post-PKS biosynthetic steps in the native/heterologous hosts. Additionally, diverse BGCs cloning techniques as Bacterial artificial chromosome (BAC) cloning, Gibson assembly, Linear–linear homologous recombination (LLHR), Golden Gate assembly and Transformation-associated recombination (TAR) have played pivotal role in unlocking diverse NP resources [146, 147] The availability of detailed genomic information of producer strains and precise analysis facilitated by efficient cloning methods and utilizing the metabolically engineered host system can be next generation approach for isolation of novel macrolides or enhanced production of existing macrolides with pharmaceutical values.

## Data Availability

Not applicable

## Abbreviations

BGCs: biosynthetic gene clusters
PKSs: polyketide synthases
NRPS: non-ribosomal peptide synthase
DEBS: 6-deoxyerythronolide B synthase
NPP: nystatin-like Pseudonocardia polyene
MRSA: methicillin resistance *Staphylococcus aureus*
MAGE: Multiplex Automated Genome Engineering
CRISPRs: Clustered Regularly Interspaced Short Palindromic Repeats
